# XG-102 administered to healthy male volunteers as a single intravenous infusion: a randomized, double-blind, placebo-controlled, dose-escalating study

**DOI:** 10.1002/prp2.20

**Published:** 2014-01-26

**Authors:** Catherine Deloche, Luis Lopez-Lazaro, Sébastien Mouz, Julien Perino, Claire Abadie, Jean-Marc Combette

**Affiliations:** 1 Solid Drug DevelopmentGeneva, Switzerland; 2 Covance Clinical Research Unit (CRU)Basel, Switzerland

**Keywords:** Clinical study, healthy volunteers, infusion, intravenous, phase I, PK, safety, tolerability, XG-102

## Abstract

The aim of the study is to evaluate the safety, tolerability and pharmacokinetics (PK) of the JNK inhibitor XG-102 in a randomized, double blind, placebo controlled, sequential ascending dose parallel group Phase 1 Study. Three groups of male subjects received as randomly assigned ascending single XG-102 doses (10, 40, and 80 *μ*g/kg; 6 subjects per dose) or placebo (2 subjects per dose) as an intravenous (IV) infusion over 60 min. Safety and tolerability were assessed by physical examination, vital signs, electrocardiography, eye examination, clinical laboratory tests and adverse events (AEs). PK was analyzed using noncompartmental methods. All reported AEs were mild to moderate and neither their number nor their distribution by System Organ Class suggest a dose relationship. Only headache and fatigue were considered probably or possibly study drug related. Headache frequency was similar for active and placebo, consequently this was not considered to be drug related but probably to study conditions. The other examinations did not show clinically relevant deviations or trends suggesting a XG-102 relationship. Geometric mean half-life was similar among doses, ranging from 0.36 to 0.65 h. Geometric mean XG-102 AUC_0–last_ increased more than linearly with dose, 90% confidence intervals (CIs) did not overlap for the two highest doses. Geometric mean dose normalized *C*_max_ values suggest a more than linear increase with dose but 90% CIs overlap. It may be concluded that XG-102 single IV doses of 10–80 *μ*g/kg administered over 1 h to healthy male subjects were safe and well tolerated.

## Introduction

**Figure 1:**
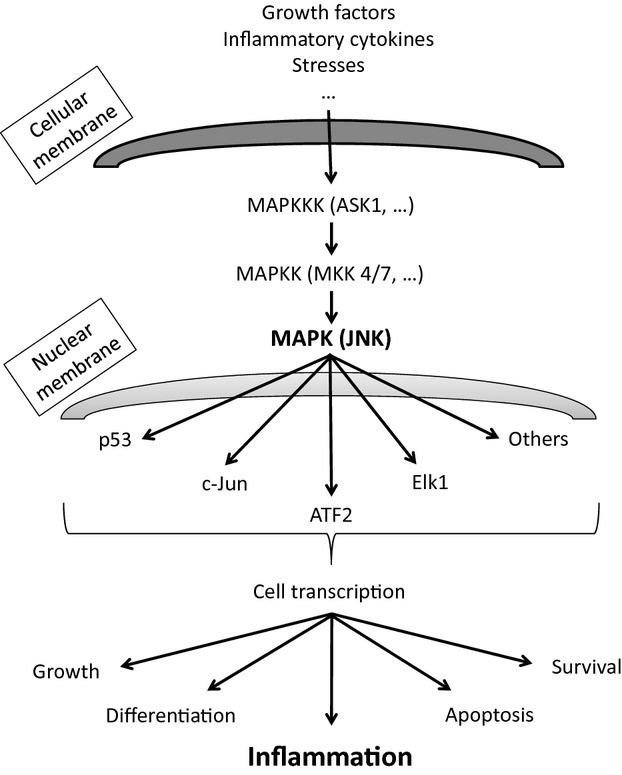
Schematic representation of JNK signaling. The JNK pathway is activated in multiple cells by various extracellular factors (including stresses and cytokines) and is involved in different cellular processes through multiple intracellular signaling. Extracellular factors lead to the activation of mitogen-activated protein kinase kinase kinases (MAPKKKs). MAPKKK activates either MAP kinase kinase 4 or 7; both MAPKK4/7 activates the JNKs MAPK. JNK activation leads to specific substrates activation and subsequent cell transcription. ASK, activator of S-phase kinase; ATF, activating transcription factor; Elk-1, member of the ETS oncogene family; JNK, c-Jun NH2-terminal kinase.

Broad cellular activities are partly mediated by mitogen-activated protein kinases (MAPKs) and their intracellular-induced signaling pathway (Kim and Choi 2010). The c-Jun N-terminal kinase 1–3 cascade, is one of the four fully elucidated MAPK cascades, which connects extracellular signals to intracellular events (Keshet and Seger 2010). The c-Jun N-terminal kinase (JNK) cascade is activated mainly by cellular stresses (Keshet and Seger 2010) including genotoxic, osmotic, hypoxic, or oxidative stress and by proinflammatory cytokines such as tumor necrosis factor (TNF)-α and interleukin (IL)-1β (Kim and Choi 2010) (Fig. 1).

The main action of JNK is essentially in the mediation of the apoptotic response of cells to proinflammatory cytokines, genotoxic and environmental stresses and its activation has been observed in medical diagnoses affecting central nervous system (CNS), cardiovascular, hepatobiliary/digestive, joint, auditory, and respiratory tissues (Sabapathy 2012), and there is evidence that transient JNK activation promotes cell survival whereas prolonged activation induces apoptosis (Davies and Tournier 2012). JNK inhibition has also been shown to decrease the formation of autophagic vesicles under conditions of endoplasmic reticulum stress (Bogoyevitch et al. 2010).

The main CNS diagnoses where JNK activation was observed include Alzheimer's disease (AD), Parkinson's disease (PD), stroke, brain contusion, brain injury, spinal cord contusion, and brain artery occlusion (Shoji et al. 2000; Zhu et al. 2001; Savage et al. 2002; Borsello et al. 2003; Zhuang et al. 2006; Colombo et al. 2009; Ortolano et al. 2009; Bessero et al. 2010; Braithwaite et al. 2010; Kim and Choi 2010; Michel-Monigadon et al. 2010; Nijboer et al. 2010; Spigolon et al. 2010; Antoniou et al. 2011; Armstead et al. 2011; Mehan et al. 2011; Sclip et al. 2011; Navon et al. 2012; Repici et al. 2012; Sabapathy 2012; Zhao et al. 2012) while the main cardiovascular conditions where JNK activation was observed include coronary events suggestive of ischemia and heart failure (Milano et al. 2007; Sabapathy 2012). In the hepatobiliary/digestive tract, JNK activation has been observed in inflammatory bowel disease (IBD), colitis, hepatic injury, liver ischemia, reperfusion injury, acute liver injury, chronic hepatitis C virus infection, and nonalcoholic fatty liver disease (Relja et al. 2009; Han et al. 2010; Reinecke et al. 2012; Sabapathy 2012; Seki et al. 2012). Other diagnoses where JNK was observed includes rheumatoid arthritis, osteoarthritis (Han et al. 1999, 2001; Guma et al. 2011) inner ear injury (Wang et al. 2003; Eshraghi et al. 2006; Omotehara et al. 2011), Behcet's disease, systemic lupus erythematosus (Sabapathy 2012), and diabetes (Bogoyevitch et al. 2010; Andreasen et al. 2011).

Given that the JNK signaling pathway is a very complex and multifactorial pathway involving many intracellular processes and stimulated by many extracellular signals, there are probably many other tissue types and inflammatory processes which have yet to be discovered that activate JNK production (Fig. 1).

**Figure 2:**
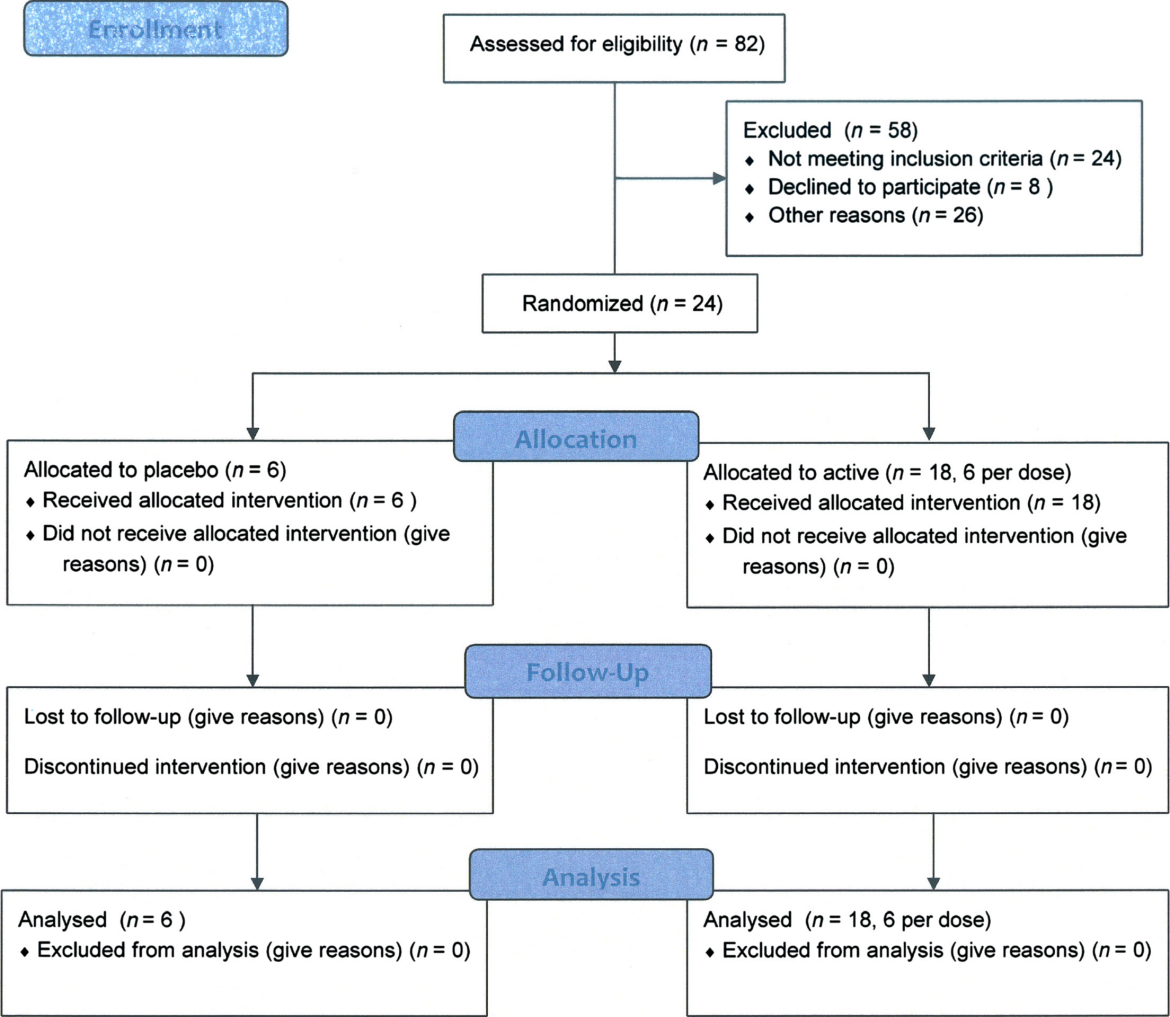
Consort 2010 flow diagram. Progress of all participants through trial execution (enrollment, allocation, follow-up, and analysis).

It has been hypothesized that JNK types may act as a mediator of cell stress responses following their role as regulators of proapoptotic death signaling events. The possible relation between JNK activation and cell death, as revealed by gene knockout and/or the use of JNK inhibitors, has stimulated the development of JNK inhibitors that can prevent cell death.

The majority of the inflammatory molecules are produced by immune cells activation caused by some non-physiological processes. Example of such molecules include TNF-α, IL-2, E-selectin, and MMP, which are regulated by the transcription factors AP-1 and ATF-2 being controlled by the JNK pathway (Manning and Davis 2003). Due to the above reasons, there may be a potential for JNK inhibition to treat specific medical conditions of inflammatory origin (Manning and Davis 2003; Keshet and Seger 2010; Kim and Choi 2010).

The discovery of the JNK-inhibitory properties of the JNK scaffold protein JIP1, followed by the identification of its minimum inhibitory sequence, has opened a new avenue in the use of JIP1-derived JNK inhibitory peptides (Bogoyevitch et al. 2010). In the program leading to the drug evaluated in this study, different peptides were obtained by linking the 19-amino acid JNK-binding motif of JIP-1 to the 10-amino acid HIV transactivator of transcription (Tat) transporter sequence. The Tat peptide is one of the cell-penetrating peptides used for intracellular drug delivery (Chen and Harrison 2007) as cell penetration capabilities are needed due to the intracellular location of the JNK signaling cascades. JIP-1 and c-Jun share a similar binding motif, but JNK's affinity of binding to JIP-1 is about 100-fold higher.

In addition to the L-form of the JNK-inhibitory peptide (L-JNKI-1) the protease-resistant all-D-retroinverso form (D-JNKI-1 with a 31 amino acids also named XG-102) was synthesized to expand its half-life in vivo. The use of D-amino acids seemed crucial, especially because the Tat sequence is containing multiple disulfide bonds (six in total) that render it extremely sensitive to the proteases involved in peptide processing in the nervous system (Borsello et al. 2003) and other organs.

XG-102 is a protease-resistant peptide composed of 31 amino acids in the D-configuration that selectively inhibits JNK activity in a non-ATP competitive manner.

The dextrogyre XG-102 peptide has been extensively studied in different pathologies as JNK was clearly demonstrated as being a key player in many inflammatory and/or pathological processes and as the peptide JNK-inhibiting function may have important therapeutic effect in such models. Among all previously cited pathologies, XG-102 demonstrated activity in all different major disease groups and among these in most of the cited JNK implicating pathologies.

Main groups wherein XG-102 demonstrated specific activity were the following (specific studied pathologies are pointed out): neuronal (AD [Braithwaite et al. 2010; Sclip et al. 2011; Colombo et al. 2009], brain ischemia [Borsello et al. 2003; Esneault et al. 2008; Benakis et al. 2010; Bessero et al. 2010; Bogoyevitch et al. 2010; Liu et al. 2010; Nijboer et al. 2010; Antoniou et al. 2011; Gow et al. 2011], seizure [Spigolon et al. 2010; Zhao et al. 2012], retinal excitotoxicity [Bessero et al. 2010], brain contusion/percussion [Ortolano et al. 2009; Armstead et al. 2011], spinal cord injury [Zhuang et al. 2006; Repici et al. 2012]), cardiovascular (myocardial ischemia reperfusion and/or ischemia [Milano et al. 2007]), digestive (IBD [Reinecke et al. 2012]), and auditive (ear injury [Wang et al. 2003; Eshraghi et al. 2006; Omotehara et al. 2011]).

In addition to the above experiments, in the rat model of uveitis induced by footpad injection of endotoxin, treatment with XG-102 by either the IV or the intravitreal route just before endotoxin administration significantly decreased the uveitis clinical scores, the numbers of infiltrating inflammatory cells and the inducible nitric oxide synthase expression in the eye as compared to vehicle (Touchard et al. 2010). Those results were a clear demonstration of the potential for treating inflammation and subsequent inflammation-related diseases with the specific XG-102 compound. Besides the impressive positive results in this ocular inflammation model, the results need to be confirmed in clinical trials demonstrating safety (phase I) and efficacy (phase II ongoing).

Human experience at the time this study was planned included the following processes:

### Intratympanic administration

A small study with AM-111 (specific XG-102 intratympanic formulation) applied intratympanically for acute acoustic trauma (firecrackers induced) in which it was safe and well tolerated but the small number of patients (*N* = 11) and the comparison limited to two AM-111 dose levels preclude conclusions on efficacy (Suckfuell et al. 2007).

A phase II study with AM-111 applied intratympanically for acute sensorineural hearing loss that was completed in October 2012 (NCT00802425).

### Systemic administration

A first-in-man study was performed in patients having sustained either a stroke or a transient ischemic attack (TIA). The study was performed as a multicenter, randomized, double-blind, placebo-controlled, single-dose escalation trial. In total, 10 patients participating in this trial were randomized in a 4:1 ratio (four active and one placebo patients). Two doses of XG-102 were evaluated in this study given in a single IV infusion. None of the patients allocated XG-102 showed signs of acute intolerance at either the start or during the study treatment infusion. The observed reactions were of different types: cardiac disorders, gastrointestinal disorders, general disorders, and administration site conditions, metabolism and nutrition disorders, skin and subcutaneous tissue disorders, vascular disorders, blood and lymphatic system disorders, ear and labyrinth disorders, musculoskeletal and connective tissue disorders, respiratory and mediastinal disorders, nervous system disorders, and investigations. None of the reported adverse events (AEs) were considered by the investigator to be related to study treatment and the severity of the majority of the reported events was considered to be mild. The AEs did not show any temporal pattern or dose-response relationship suggesting a relationship to XG-102 but this affirmation has to be read within the constraints of the limited available sample size (Unpublished data on file, Xigen SA).

### Subconjunctival administration

A phase Ib with XG-102 administered subconjunctivally in patients with post surgery or post traumatic intraocular inflammation was conducted as a monocenter, open-labeled, multiple-dose escalation trial. A total of 20 patients were included in the study, divided into four groups (one dose per group was tested given in a single subconjunctival injection) of five patients. Patients were followed up for 4 weeks to collect safety and tolerance data. Patients' follow-up was 100%. None of the patients allocated XG-102 showed signs of intolerance at either time during the study and observed AEs (different types of reactions were observed: blood and lymphatic system disorders, general disorders and administration site conditions, ocular disorders, and investigations) were not attributed to drug study and for those existing they were rated as mild or moderate degree (for two of them) [Unpublished data on file, Xigen SA].

A phase II is currently ongoing with XG-102 administered subconjunctivally in patients with post surgery inflammation. This study was designed as a multicenter, double-blind, controlled study. A total of 138 patients were included in three groups: two groups receive a single subconjunctival injection of XG-102 (two doses) and placebo eye drops four times a day for 21 days, and the third group receives a single subconjunctival injection of placebo and dexamethasone eye drops four times a day for 21 days.

In this publication, we describe the result of a phase I study where 24 healthy subjects received 60-min intravenous infusion of XG-102 or placebo (Fig. 2).

## Methods

### Ethics statement

The study protocol, the informed consent form (ICF) used to obtain the consent of the study subjects and the study advertising materials were approved before study start by both the Ethics Committee of Both Basels (EKBB) and Swissmedic (institute regulating drugs and drug research in Switzerland). This study was registered in ClinicalTrials.gov with Identifier NCT01570205.

### Methods

This was a single-center, randomized, double-blind, placebo-controlled (parallel treatment within dose groups), ascending single-dose, sequential group study. Three separate groups of eight subjects were studied in ascending order of dose. In each group, six subjects received XG-102 and two subjects received placebo. Each subject received a single IV dose of either XG-102 or placebo during the study. The study sample size was based on empirical considerations and in the numbers usually sufficient to fulfill the objectives of this type of study, no statistical power based calculation was made.

For each dose group, one subject was treated at a time; if administration was well tolerated, and if no significant AEs occurred, the next subject was treated no less than 24 h later. There was an interval of at least 10 days between each dose group, to allow a satisfactory review of the safety and pharmacokinetics (PK) data from the lower doses prior to progression to the next higher dose.

The only protocol amendment was written before the study start, no major changes in methods occurred during the study conduct.

Written informed consent was obtained from potential subjects by a study physician at the screening visit using the approved ICF before any assessments were undertaken. It was emphasized that the volunteer was at liberty to withdraw their consent to participate at any time, without penalty or loss of benefits to which the volunteer was otherwise entitled. Volunteers who refused to give, or withdrew written informed consent were not included or continued in this study.

To be eligible, subjects had to be of male gender, aged between 18 and 45 years inclusive, with a body mass index (BMI) between 18.5 and 30.0 kg/m^2^ inclusive, and determined to be healthy by a screening examination including demographic data, medical history, physical examination, body temperature, sitting vital signs, 12-lead electrocardiogram, fundoscopy, intraocular pressure (IOP) measurement, a laboratory hematology examination including hemoglobin, hematocrit, and complete cell counts with differential, serum clinical chemistry, urine dipstick examination, and serology for hepatitis B and C, HIV, and tuberculosis.

The informed consent was obtained and the screening examinations were performed in the designated area for outpatient visits of the Covance Basel Research Unit (CBRU).

### Treatment assignment and assignment concealing

Each eligible subject was allocated a numbered study treatment pack according to the order of attendance to CBRU. Volunteers were randomized using SAS (SAS Institute, Cary NC) version 9.1 within each dose group in a 3:1 ratio to XG-102 or placebo. By study cohort, two blocks of four subjects were randomized (three to the corresponding XG-102 dose; one to the placebo). Replacement subjects were similarly randomized.

The random allocation sequence was generated by the CBRU Statistics Department.

The study subjects and all CBRU personnel caring for or evaluating the study subjects were blinded to the treatment assignment.

The following controls were employed to maintain the double-blind status of the study:

1. Placebo vials and their contents were identical in appearance to the vials containing XG 102.
2. The investigator and other members of staff involved with the study remained blinded to the treatment randomization during the whole study.

The study pharmacist was unblinded and prepared the study medication corresponding to each subject. The study medication was later provided to the Investigational team without any indication of the treatment identity.

To enable the investigator to break the code, if required for safety reasons, individual sealed envelopes containing the treatment code for each subject were kept in CBRU.

### Interventions

For each volunteer, 2 mL was withdrawn from the study product vial allocated to him and added to a syringe containing 28 mL of 0.9% sodium chloride solution. Three different concentrations of product (2 mL of 0.75, 3, and 6 mg/mL before dilution) were used, hence concentrations used for infusion after dilution (50, 200 and 400 μg/mL) defined the specific patient treatment groups (10, 40, 80 μg/kg). The XG-102 doses were intravenously infused in a peripheral vein from the arm at final concentrations between 50 and 400 μg/mL depending on the dose group for 60 min with a calibrated syringe pump. Subjects were dosed in numerical order according to the treatment randomization. Dosing occurred in CBRU at similar times for all groups, commencing between 08:30 and 08:45.

### Outcome measures

Safety and tolerability were assessed on the basis of the following evaluations: Physical examination, vital signs (blood pressure [BP], pulse rate [PR]), 12-lead electrocardiography (ECG) and continuous monitoring of ECG rhythm (V2 lead) for the first 4 h after dosing, fundus of the eye (nondilatated pupil), IOP, clinical laboratory tests (hematology, coagulation, serum chemistry, and urinalysis), AEs, and assessment of tolerability by investigator.

The condition of each subject was monitored throughout the study. Subjects were required to report any AEs during the study. The nature, time of onset, duration, and severity were documented, together with the investigator's opinion of the relationship to drug administration. Subjects were followed up in the study unit for the 24 h after the administration of the study drug and came back to the unit for two follow-up visits 8 ± 2 and 28 ± 5 days after dosing. All study visits and activities were performed in BCRU.

Physical examination was performed at screening and day 8 of follow-up. Vital signs, body weight, and 12-lead ECG were evaluated at screening, before dosing, before discharge from the Unit, and at both follow-up visits. Continuing ECG monitoring with a single lead (V2) was done for the first 4 h after the start of study drug infusion and sitting BP, PR, and rhythm measurement were recorded at 5, 15, 30, 45 min, and 2 h after the start of infusion. Fundoscopy and IOP measurement were done at screening, once during the day after dosing and at the day 8 follow-up visit. Clinical chemistry, coagulation, laboratory hematology, and dipstick urine analysis were obtained at screening, 1 and 24 h after start of infusion, and at both follow-up visits. AEs could be volunteered by the subjects at any time.

For quantification of XG-102 plasma concentrations, 2 mL blood samples were taken in lithium heparin tubes from the arm not used for drug infusion at the following time points: 60, 65, 70, 75, 90, and 110 min, 2, 3, 4, 5, 7, 9, 11, 13, 16, and 24 h after the start of the study drug infusion. No samples for PK were taken from the IV line used for study treatment infusion.

For quantification of the infused dose of XG-102, two 0.5 mL aliquots of the study treatment infusate were taken after infusion from the tubing at the end of the syringe.

### Analytical methods

XG-102 concentrations in plasma samples were determined by a validated high-performance liquid chromatography, tandem mass spectrometry assay with a lower limit of quantification (LLOQ) of 10 ng/mL; this analytical method is considered precise, accurate, and specific with the following characteristics: intra-precision (1.4–18.8 acceptable because it is concerning the LLOQ), inter-precision (5.7–16.8 acceptable because it is concerning the LLOQ); intra-accuracy (91–100%), inter-accuracy (100–113%); and specificity (no interference <5%). Quantification of XG-102 in the infusate was performed using a validated HPLC-ultraviolet assay with a LLOQ of 1 μg/mL is also defined by the following characteristics: intra-precision (0.7–3.7), inter-precision (3.3–6.3); intra-accuracy (96.6–107%), inter-accuracy (95.4–113%); and specificity (no interference <5%).

The pharmacokinetic analysis was conducted by BCRU using WinNonlin Professional Version 4.1 (Pharsight Corporation, Mountain View, CA).

Pharmacokinetic parameters were determined from the plasma concentrations of XG-102 using noncompartmental procedures. The PK parameters determined are presented in Table 1.

**Table 1:** Pharmacokinetic parameters determined for XG-102

Parameter	Definition
*C*_max_	Maximum measured concentration of the analyte in plasma
CL	Clearance
*t*_max_	Time from dosing to maximum measured concentration
AUC_0–∞_	Area under the concentration-time curve of the analyte in plasma over the time interval from 0 extrapolated to infinity
AUC_0–last_	Area under the concentration-time curve of the analyte in plasma over the time interval from 0 up to the last quantifiable plasma concentration
%AUC_extrap_	Percentage of AUC_0–∞_ extrapolated beyond the last quantifiable plasma concentration
*λ*_*z*_	Terminal rate constant in plasma
*t*_½_	Terminal half-life of the analyte in plasma
MRT	Mean residence time
*V*_ss_	Volume of distribution at steady state

AUC_0–last_ (area under the curve) was calculated using the linear log-trapezoidal method.

### Statistical analysis

The planned sample size is not based on a power calculation. The resulting group sizes (six on active, two on placebo) are in general considered as sufficient for this type of study.

Three analysis populations were defined–the intention-to-treat (ITT) analysis set, the per protocol (PP) analysis set, and the pharmacokinetic analysis set. The ITT analysis population consisted of all volunteers in whom the study medication infusion was started. The PP analysis population consisted of all volunteers who did not have any major violations with the inclusion and exclusion criteria, who received the study medication infusion for at least 45 min and in whom all follow-up visits and assessments were complete. The pharmacokinetic population consisted of all subjects who received one dose of study drug and had evaluable PK data. For the safety analysis, volunteers were analyzed as treated.

AEs were coded using the Medical Dictionary for Drug Regulatory Activities (MedDRA) version 14.1. AEs occurring up to 8 days after the start of study treatment administration were assigned to the treatment period for evaluation. Independent of this rule, the relationship of an AE to the study treatments was assessed by the investigator.

Listings of all reported AEs were presented. Descriptive analyses of AEs were also performed. Incidence was reported by frequency and body system for each dose group.

All evaluable subjects were included in the pharmacokinetic analysis. Subjects who were considered as not evaluable were listed with their individual plasma concentrations and PK parameters, however, were not included in the descriptive statistics for plasma concentrations, PK parameters or other statistical assessment.

For PK parameters, descriptive statistics were tabulated by dose. For *t*_max_, due to its discrete numerical distribution, only minimum, median, and maximum values were included in the tabulation.

The PK parameters, *C*_max_, AUC_0–last_, and AUC_0–∞_ were dose normalized by dividing them by the dose. Descriptive statistics, including conventional 90% confidence intervals (CI) for the arithmetic and geometric mean at each dose were exploratorily generated.

Descriptive statistics for the study data were determined using SAS® Version 9.1 (SAS Institute).

## Results

### Subject disposition

The clinical conduct of the study started on 21 July 2011 and finished on 8 November 2011. The study ended when all scheduled subjects at each protocol scheduled dose cohort had been treated and completed their follow-up as stipulated by the protocol.

Twenty-four subjects were randomized and entered the study. All subjects completed the study in accordance with the protocol and the treatment randomization and were included in all analysis populations for all protocol defined outcomes.

All subjects were men of Western European descent aged between 18 and 45 years. Screening demographic data for subjects receiving each of the study treatments are summarized in Table 2.

**Table 2:** Summary of screening demographic data

Treatment	Placebo	10 μg/kg XG-102	40 μg/kg XG-102	80 μg/kg XG-102
Number of subjects	6	6	6	6
Age (years)	34 (8.7)	36 (4.8)	29 (9.8)	36 (9.3)
BMI (kg/m^2^)	22.5 (2.15)	24.7 (1.97)	22.3 (1.82)	24.6 (1.76)

Arithmetic mean (standard deviation [SD]) data are presented.

### Outcomes

#### Safety and tolerability

The overall incidence of treatment emergent AEs is summarized in Table 3.

**Table 3:** Summary of treatment emergent adverse events

XG-102 Dose	Placebo	10 μg/kg	40 μg/kg	80 μg/kg
Number of subjects (%) with adverse events [number of adverse events]				
Number of subjects treated	6	6	6	6
All treatment emergent adverse events				
Mild	1 (16.7%) [2]	2 (33.3%) [2]	4 (66.7%) [4]	2 (33.3%) [3]
Moderate	1 (16.7%) [1]	1 (16.7%) [3]	1 (16.7%) [1]	0
Severe	0	0	0	0
Total	2 (33.3%) [3]	3 (50.0%) [5]	5 (83.3%) [5]	2 (33.3%) [3]
Possibly, probably, or definitely related adverse events				
Mild	1 (16.7%) [1]	0	1 (16.7%) [1]	1 (16.7%) [1]
Moderate	1 (16.7%) [1]	0	1 (16.7%) [1]	0
Severe	0	0	0	0
Total	2 (33.3%) [2]	0	2 (33.3%) [2]	1 (16.7%) [1]
Subjects discontinued due to adverse events				
Total	0	0	0	0
Subjects with serious adverse events				
Total	0	0	0	0

None of the subjects experienced serious or severe AEs, and none of the subjects were discontinued due to AEs.

All treatment emergent AEs are summarized by frequency in Table 4.

**Table 4:** Frequency of treatment emergent adverse events (all causalities)

XG-102 dose	Placebo	10 μg/kg	40 μg/kg	80 μg/kg
Number of subjects treated	6	6	6	6
*MedDRA Preferred Term by System Organ Class*	Number of subjects with adverse events [number of adverse events]			
Nervous system disorders				
Headache	2 [2]		3 [3]	
Total	2 [2]		3 [3]	
Gastrointestinal disorders				
Diarrhea		2 [2]		
Nausea			1 [1]	
Total		2 [2]	1 [1]	
General disorders and administration site conditions				
Fatigue				1 [1]
Local swelling				1 [1]
Total				2 [2]
Ear and labyrinth disorders				
Otitis media		1 [1]		
Total		1 [1]		
Infection and Infestations				
Nasopharyngitis	1 [1]			
Total	1 [1]			
Injury, poisoning, and procedural complications				
Contusion		1 [1]		
Total		1 [1]		
Respiratory, thoracic and mediastinal disorders				
Chest pain			1 [1]	
Total			1 [1]	
Skin injuries				
Laceration		1 [1]		
Total		1 [1]		
Vascular disorders				
Hematoma				1 [1]
Total				1 [1]
Overall total	2 [3]	3 [5]	4 [5]	2 [3]

Treatment emergent AEs that were considered related to administration of the study drug are summarized by frequency in Table 5.

**Table 5:** Frequency of treatment emergent adverse events (drug-related)

XG-102 dose	Placebo	10 μg/kg		40 μg/kg	80 μg/kg
Number of subjects treated	6	6		6	6
*MedDRA Preferred Term by System Organ Class*	Number of subjects with adverse events [number of adverse events]				
Nervous system disorders					
Headache	2 [2]			2 [2]	
Total	2 [2]			2 [2]	
General disorders and administration site conditions					
Fatigue					1 [1]
Total					1 [1]
Overall total	2 [2]			2 [2]	1 [1]

There were no treatment or dose-related trends in the serum chemistry, hematology, coagulation or urinalysis parameters during the study. Results for some clinical laboratory parameters were outside their reference ranges, but these findings were generally transient and occurred at isolated time points only. No findings were considered to be of clinical importance.

There were no clinically significant findings in the physical examination. Likewise, there were no treatment or dose-related trends in sitting systolic and diastolic BP, PR or body temperature. Transient changes in BP and PR were noted at isolated time points for some subjects, but none of these findings were considered clinically important.

There were no clinically significant findings in cardiac monitoring and no apparent treatment or dose-related trends in the 12-lead ECG parameters were noted. In particular, there was no evidence of prolongation of corrected QT interval (using both the Fridericia and the Bazett correction) at each dose of XG–102. There were no clinically important findings in the 12-lead ECG morphology for any subject. Likewise, there were no clinically significant findings in IOP or fundus examinations.

Due to these reasons, no further details are presented on the serum biochemistry, hematology, coagulation, urinalysis, physical examination, vital signs, ECG examinations, IOP, and fundus examinations during the study.

Concomitant medication was used by three subjects during the study. One subject treated with 40 μg/kg XG-102 received a single oral 500 mg paracetamol dose on day 1 for a headache. One subject treated with 10 μg/kg XG-102 received, a 4 mL subcutaneous prilocaine hydrochloride dose on day 3 for a cut to his right thumb. Approximately 3 weeks after dosing this same subject received 14 mg oral ciprofloxacin hydrochloride for otitis media, and albumin tannate for diarrhea. Approximately 1 month after dosing, a subject treated with 80 μg/kg XG-102 used topical heparin, as needed, for a bulge at the back of the head.

#### Pharmacokinetics

XG-102 plasma concentrations were below the LOQ by at most 2, 3, and 7 h after the start of 10, 40, and 80 μg/kg IV infusions, respectively.

The PK parameters of XG-102 are summarized in Table 6. The plasma *C*_max_, AUC_0–last_, and AUC_0–∞_ of XG-102 increased with dose. Extrapolated AUC exceeded 20% in most subjects treated with 10 μg/kg (median value 34.1% as shown in Table 6). Hence, the evaluation of AUC_0–∞_ and CL values has to be viewed as exploratory.

**Table 6:** Summary of the pharmacokinetic parameters for XG-102 following single intravenous doses administered as a constant rate infusion over 1 h

XG-102 dose	10 μg/kg	40 μg/kg	80 μg/kg
Number of subjects	6	6	6
Parameter			
AUC_0–last_ (ng h/mL)	24.7 (20.0–30.5)	134 (118–152)	431 (312–596)
AUC_0–∞_ (ng h/mL)	36.8 (30.5–44.6)	146 (126–168)	443 (320–612)
AUC_extrap_^1^ (%)	34.1 (18.6–49.7)	6.7 (4.2–12.9)	2.9 (1.9–3.4)
*C*_max_ (ng/mL)	31.3 (25.7–38.2)	146 (128–168)	362 (274–479)
*t*_max_^1^(h)	1.00 (1.00–1.05)	1.00 (1.00–1.00)	1.00 (1.00–1.00)
MRT (h)	1.00 (0.79–1.27)	0.76 (0.70–0.84)	1.02 (0.91–1.15)
*t*_½_ (h)	0.57 (0.40–0.81)	0.36 (0.30–0.43)	0.65 (0.48–0.89)
CL (mL/h)	17537 (14441–21297)	18399 (16092–21037)	13217 (9381–18621)
CL (mL/h/kg)	217 (177.3–264.8)	253 (222.1–287.4)	165 (118.4–229.1)
*V*_ss_ (mL)	17536 (13078–23513)	14040 (12346–15967)	13500 (10564–17251)
*V*_ss_ (mL/kg)	217 (174–271)	193 (172–216)	168 (132–214)

Geometric mean (90% confidence limits) data are presented.

1 Median (minimum–maximum).

Dose proportionality is explored in Table 7. The dose-normalized pharmacokinetic parameter values shown in Table 7 suggest that *C*_max,_ AUC_0–last,_ and exploratorily AUC_0–∞_ increase more than in proportion with XG-102 dose in the evaluated dose range.

**Table 7:** Evaluation of XG-102 pharmacokinetic parameters dose proportionality following single intravenous doses administered as a constant rate infusion over 1 h

XG-102 dose	10 μg/kg	40 μg/kg	80 μg/kg
Number of subjects	6	6	6
Parameter			
AUC_0–last_(norm) (ng h/mL)/(μg/kg)	3.10 (2.44–3.92)	3.64 (3.25–4.08)	5.91 (4.25–8.22)
AUC_0–∞_(norm) (ng.h/mL)/(μg/kg)	4.61 (3.78–5.64)	3.96 (3.48–4.50)	6.07 (4.36–8.44)
*C*_max_(norm) (ng/mL)/(μg/kg)	3.93 (3.13–4.92)	3.98 (3.49–4.53)	4.97 (3.74–6.60)

Geometric mean (90% CI) data are presented. (norm) = normalized for dose and body weight.

The 90% CIs for geometric mean dose-normalized AUC_0–last_ are higher for the 80 μg/kg dose and do not overlap with those for the other doses. For the 10 and 40 μg/kg doses, 90% CIs for the geometric mean dose-normalized AUC_0–last_ overlap, but the geometric mean after the 10 μg/kg dose is lower than and outside the 90% CI for the geometric mean dose-normalized AUC_0–last_ after the 40 μg/kg dose.

The 90% CIs for the geometric mean dose-normalized *C*_max_ widely overlap between the 10 and 40 μg/kg doses, but only limitedly overlap between the 40 and 80 μg/kg doses. The geometric mean dose-normalized *C*_max_ for the 80 μg/kg dose is higher than and outside the 90% CIs for geometric mean dose-normalized *C*_max_ for the other doses.

The intersubject variability in *C*_max_ after 10 and 40 μg/kg doses is moderate (geometric mean CV% in the 16.7% to 24.4% range [data not shown]). The intersubject variability in AUC_0–last_ and AUC_0–∞_ is similarly moderate after 10 and 40 μg/kg doses (geometric mean CV% ranging from 15.2 to 26.1% for AUC_0–last_ and from 17.5% to 23.4% for AUC_0–∞_). However, after the highest 80 μg/kg dose, the intersubject variability is higher (geometric mean CV% 34.9% for *C*_max_; 41.0% for both AUC_0–last_ and AUC_0–∞_ [data not shown]).

After the end of infusion, XG-102 plasma concentrations quickly declined. Geometric mean *t*_1/2_ per dose ranged from 0.36 to 0.65 h and geometric mean mean residence time (MRT) per dose ranged from 0.76 to 1.02 h (Table 6). The variability was higher for *t*_1/2_ (geometric mean CV% per dose ranging from 22.3% to 44.6%) than for MRT (geometric mean CV% per dose ranging from 11.0% to 29.9%). Geometric mean *t*_1/2_ and MRT did not clearly differ among doses (Table 6).

Geometric mean CL per dose was in the 17537–18399 mL/h range after 10 and 40 μg/kg doses and lower (13217 mL/h) after 80 μg/kg doses. The body weight normalized geometric mean CL per dose was in the 217–253 mL/h/kg range after 10 and 40 μg/kg doses and lower (165 mL/h/kg) after 80 μg/kg doses.

The intersubject variability in CL was moderate after doses of 10 and 40 μg/kg (geometric mean CV% for total CL of 23.9% and 16.4% after 10 and 40 μg/kg, respectively; geometric mean CV% for body weight normalized CL of 24.8% and 15.7% after 10 and 40 μg/kg, respectively). After the highest 80 μg/kg dose, the variability in CL was higher (geometric mean CV% of 43.5% for total body CL; geometric mean CV% of 41.8% for body weight-normalized CL).

The geometric mean *V*_ss_ was in the 14040–17536 mL range after 40 and 10 μg/kg doses, and lower (13500 mL) after 80 μg/kg dose. The body weight-normalized geometric mean *V*_ss_ was in the 193–217 mL/kg range after 40 and 10 μg/kg doses and lower (168 mL/kg) after 80 μg/kg dose.

The intersubject variability in *V*_ss_ was moderate, being slightly higher for *V*_ss_ (geometric mean CV% for total *V*_ss_ of 36.8%, 15.7%, and 30.5% after 10, 40, and 80 μg/kg, respectively; geometric mean CV% values for body weight normalized *V*_ss_ of 27.5%, 13.7%, and 29.8% after 10, 40, and 80 μg/kg, respectively).

## Discussion and Conclusions

Safety and tolerability were assessed by reported AEs, physical examination, ECG, vital signs, ophthalmic examination (fundoscopy and IOP measurement), clinical laboratory tests (serum chemistry, coagulation, laboratory hematology, and urine analysis).

Only mild to moderate AEs were reported. No serious AEs were reported. Neither the number of reported AEs in each System Organ Class nor the number of reports for each individual AE suggest a dose-response relationship to XG-102 (Tables 3, 4).

Five AEs, reported by five subjects, were considered by the investigator to be probably or possibly related to the study drug, and of these five subjects, two had received placebo (Table 5).

During blinded causality assignments, headache was considered related to the study drug. Upon unblinding, headache frequency was found to be similar for XG-102 and placebo and no headache was reported with the highest 80 μg/kg XG-102 dose. Fatigue was the only other AE considered to have a possible or probable relationship to the study drug. Fatigue was reported by one subject (treated with 80 μg/kg XG-102), at 30 min post dose, but it was graded as mild and resolved without treatment after 15 min (Table 5) (there was no clinically significant finding in vital signs at the same time).

Clinical laboratory examinations, vital signs, and ECG including cardiac monitoring evaluations did not show any clinically relevant deviation from their normal ranges or numerical trends with a dose-response relationship or temporal pattern which may suggest a relationship to the administration of XG-102.

In the XG-102 PK evaluation, XG-102 body weight-normalized V_ss_ (geometric mean ranging from 168 to 217 mL/kg in the different doses [Table 6]) is similar or slightly higher (13.7–29.8%) than the expected value of 19% of total body weight for extracellular water and higher than the expected values of 4% and 8% of body weight expected for the plasma and the blood volume, respectively (Gibaldi 1991) suggesting a relatively wide extravascular distribution.

The geometric mean by dose of *t*_1/2_ and MRT ranged from 0.36 to 0.65 h and from 0.76 to 1.02 h (Table 6), respectively, showing a rapid disappearance of XG-102 from the plasma. MRT and *t*_1/2_ do not clearly differ among doses. The results of this study do not allow deciding whether this rapid t_1/2_ corresponds to an initial phase or to the actual terminal phase of the PK profile.

XG-102 *C*_max_ and AUC (AUC_0–last_ and AUC_0–∞_) increased with dose. The geometric mean values with 90% CIs for dose-normalized AUC_0–last_ (Table 7), demonstrate a more than linear increase in AUC_0–last_ with dose (no overlap in the 90% CIs) in the 40–80 μg/kg range. Data are suggestive but inconclusive for the 10–40 μg/kg dose range (overlapping 90% CIs, but the geometric mean dose-normalized AUC_0–last_ after 10 μg/kg is lower than after 40 μg/kg).

For dose-normalized *C*_max_, all 90% CIs for the geometric mean after the different doses overlap. The geometric mean dose-normalized *C*_max_ for the 80 μg/kg dose is higher than and outside the 90% CIs for the geometric mean dose-normalized *C*_max_ for the other doses, suggesting that there may be an increasing trend in *C*_max_/dose, which is not conclusively demonstrated due to the small sample size (six subjects per dose).

The intersubject variability in the PK parameters is moderate after 10 and 40 μg/kg doses (geometric mean CV% for most parameters approximately in the 15–30% range, exceptions are *t*_1/2_ and nonbody weight-normalized *V*_ss_ at the lower dose, the latter potentially due to the short quantitation period). In subjects treated with the highest 80 μg/kg dose, intersubject variability was higher, approximately in the 29–44% range, other than for MRT. This higher variability may be an effect of the low sample size or of the observed nonlinearities.

The geometric mean CV% of body weight and nonbody weight-normalized CL and *V*_ss_ are similar, suggesting that body weight normalization of the XG-102 dose had limited efficacy in decreasing the interindividual variability in exposure. However, the study design and the available sample size only allow a tentative evaluation of this issue.

The study population features, summarized in Table 2, and the subject compliance with all inclusion and exclusion criteria makes unlikely that baseline subject condition has had an undue influence in the study results. Likewise, the administered previous and concomitant medications would not be expected to interfere with the study medication and its evaluation.

This study evaluated the safety, tolerability, and PK of XG-102 in male healthy subjects, who were all of Western European descent. Due to these reasons, the applicability of the data shown here to other populations (female subjects, subjects from other racial origin, patient populations…) cannot be assumed and has to be evaluated by future studies.

Regarding PK, it may be concluded that after the end of XG-102 IV infusion, plasma concentrations quickly decreased, as shown by a short *t*_1/2_ (geometric mean ranging from 0.36 to 0.65 h at the different doses for *t*_1/2_). XG-102 AUC_0–last_ increased in a more than linear proportion with dose, with nonoverlapping 90% CI for the geometric mean dose-normalized AUC_0–last_ between the 40 and 80 μg/kg doses and only limited overlap between the 10 and 40 μg/kg doses. XG-102 *C*_max_ also appeared to increase in a more than linear proportion with dose but results are inconclusive as the geometric mean dose-normalized *C*_max_ for the 80 μg/kg dose higher than and outside the 90% CI for the other dose groups, but the 90% CIs overlap among all dose levels.

We can conclude from this study that XG-102 was safe and well tolerated when administered as single IV doses of 10–80 μg/kg over 1 h to healthy male subjects. XG-102 pharmacokinetics was evaluated with a geometric mean half-life of XG-102 in plasma ranging from 0.36 to 0.65 H. The incidence of AEs in subjects who received XG-102 or placebo was similar. No trends suggesting a relationship to XG-102 dose were apparent for any AE. All AEs observed during the study were mild to moderate with no specific dose relationship. There were no clinically significant findings in clinical laboratory data, vital signs, ECGs, physical examinations or ocular examinations (fundus and IOP).

## References

[b1] Andreasen AS, Kelly M, Berg RM, Møller K, Pedersen BK (2011). Type 2 diabetes is associated with altered NF-κB DNA binding activity, JNK phosphorylation, and AMPK phosphorylation in skeletal muscle after LPS. PLoS ONE.

[b2] Antoniou X, Falconi M, Di Marino D, Borsello T (2011). JNK3 as a therapeutic target for neurodegenerative diseases. J Alzheimers Dis.

[b3] Armstead WM, Kiessling JW, Riley J, Cines DB, Higazi AA (2011). tPA contributes to impaired NMDA cerebrovasodilation after traumatic brain injury through activation of JNK MAPK. Neurol Res.

[b4] Benakis C, Bonny C, Hirt L (2010). JNK inhibition and inflammation after cerebral ischemia. Brain Behav Immun.

[b5] Bessero AC, Chiodini F, Rungger-Brändle E, Bonny C, Clarke PG (2010). Role of the c-Jun N-terminal kinase pathway in retinal excitotoxicity, and neuroprotection by its inhibition. J Neurochem.

[b6] Bogoyevitch MA, Ngoei KR, Zhao TT, Yeap YY, Ng DC (2010). c-Jun N-terminal kinase (JNK) signaling: recent advances and challenges. Biochim Biophys Acta.

[b7] Borsello T, Clarke PG, Hirt L, Vercelli A, Repici M, Schorderet DF (2003). A peptide inhibitor of c-Jun N-terminal kinase protects against excitotoxicity and cerebral ischemia. Nat Med.

[b8] Braithwaite SP, Schmid RS, He DN, Sung ML, Cho S, Resnick L (2010). Inhibition of c-Jun kinase provides neuroprotection in a model of Alzheimer's disease. Neurobiol Dis.

[b9] Chen L, Harrison SD (2007). Cell-penetrating peptides in drug development: enabling intracellular targets. Biochem Soc Trans.

[b10] Colombo A, Bastone A, Ploia C, Sclip A, Salmona M, Forloni G (2009). JNK regulates APP cleavage and degradation in a model of Alzheimer's disease. Neurobiol Dis.

[b11] Davies C, Tournier C (2012). Exploring the function of the JNK (c-Jun N-terminal kinase) signalling pathway in physiological and pathological processes to design novel therapeutic strategies. Biochem Soc Trans.

[b12] Eshraghi AA, He J, Mou CH, Polak M, Zine A, Bonny C (2006). D-JNKI-1 treatment prevents the progression of hearing loss in a model of cochlear implantation trauma. Otol Neurotol.

[b13] Esneault E, Castagne V, Moser P, Bonny C, Bernaudin M (2008). D-JNKi, a peptide inhibitor of c-Jun N-terminal kinase, promotes functional recovery after transient focal cerebral ischemia in rats. Neuroscience.

[b14] Gibaldi M, Gibaldi M (1991). Drug Disposition. – Distribution. Biopharmaceutics and clinical pharmacokinetics.

[b15] Gow WR, Campbell K, Meade AJ, Watt PM, Milech N, Knuckey NW (2011). Lack of neuroprotection of inhibitory peptides targeting Jun/JNK after transient focal cerebral ischemia in spontaneously hypertensive rats. J Cereb Blood Flow Metab.

[b16] Guma M, Ronacher LM, Firestein GS, Karin M, Corr M (2011). JNK-1 deficiency limits macrophage-mediated antigen-induced arthritis. Arthritis Rheum.

[b17] Han Z, Boyle DL, Aupperle KR, Bennett B, Manning AM, Firestein GS (1999). Jun N-terminal kinase in rheumatoid arthritis. J Pharmacol Exp Ther.

[b18] Han Z, Boyle DL, Chang L, Bennett B, Karin M, Yang L (2001). c-Jun N-terminal kinase is required for metalloproteinase expression and joint destruction in inflammatory arthritis. J Clin Invest.

[b19] Han D, Shinohara M, Ybanez MD, Saberi B, Kaplowitz N (2010). Signal transduction pathways involved in drug-induced liver injury. Handb Exp Pharmacol.

[b20] Keshet Y, Seger R (2010). The MAP kinase signaling cascades: a system of hundreds of components regulates a diverse array of physiological functions. Methods Mol Biol.

[b21] Kim EK, Choi EJ (2010). Pathological roles of MAPK signaling pathways in human diseases. Biochim Biophys Acta.

[b22] Liu JR, Zhao Y, Patzer A, Staak N, Boehm R, Deuschl G (2010). The c-Jun N-terminal kinase (JNK) inhibitor XG-102 enhances the neuroprotection of hyperbaric oxygen after cerebral ischaemia in adult rats. Neuropathol Appl Neurobiol.

[b23] Manning AM, Davis RJ (2003). Targeting JNK for therapeutic benefit: from junk to gold?. Nat Rev Drug Discov.

[b24] Mehan S, Meena H, Sharma D, Sankhla R (2011). JNK: a stress-activated protein kinase therapeutic strategies and involvement in Alzheimer's and various neurodegenerative abnormalities. J Mol Neurosci.

[b25] Michel-Monigadon D, Bonny C, Hirt L (2010). c-Jun N-terminal kinase pathway inhibition in intracerebral hemorrhage. Cerebrovasc Dis.

[b26] Milano G, Morel S, Bonny C, Samaja M, von Segesser LK, Nicod P (2007). A peptide inhibitor of c-Jun NH2-terminal kinase reduces myocardial ischemia-reperfusion injury and infarct size in vivo. Am J Physiol Heart Circ Physiol.

[b27] Navon H, Bromberg Y, Sperling O, Shani E (2012). Neuroprotection by NMDA preconditioning against glutamate cytotoxicity is mediated through activation of ERK 1/2, inactivation of JNK, and by prevention of glutamate-induced CREB inactivation. J Mol Neurosci.

[b28] Nijboer CH, van der Kooij MA, van Bel F, Ohl F, Heijnen CJ, Kavelaars A (2010). Inhibition of the JNK/AP-1 pathway reduces neuronal death and improves behavioral outcome after neonatal hypoxic-ischemic brain injury. Brain Behav Immun.

[b29] Omotehara Y, Hakuba N, Hato N, Okada M, Gyo K (2011). Protection against ischemic cochlear damage by intratympanic administration of AM-111. Otol Neurotol.

[b30] Ortolano F, Colombo A, Zanier ER, Sclip A, Longhi L, Perego C (2009). c-Jun N-terminal kinase pathway activation in human and experimental cerebral contusion. J Neuropathol Exp Neurol.

[b31] Reinecke K, Eminel S, Dierck F, Roessner W, Kersting S, Chromik AM (2012). The JNK inhibitor XG-102 protects against TNBS-induced colitis. PLoS ONE.

[b32] Relja B, Schwestka B, Lee VS, Henrich D, Czerny C, Borsello T (2009). Inhibition of c-Jun N-terminal kinase after hemorrhage but before resuscitation mitigates hepatic damage and inflammatory response in male rats. Shock.

[b33] Repici M, Chen X, Morel MP, Doulazmi M, Sclip A, Cannaya V (2012). Specific inhibition of the JNK pathway promotes locomotor recovery and neuroprotection after mouse spinal cord injury. Neurobiol Dis.

[b34] Sabapathy K (2012). Role of the JNK pathway in human diseases. Prog Mol Biol Transl Sci.

[b35] Savage MJ, Lin YG, Ciallella JR, Flood DG, Scott RW (2002). Activation of c-Jun N-terminal kinase and p38 in an Alzheimer's disease model is associated with amyloid deposition. J Neurosci.

[b36] Sclip A, Antoniou X, Colombo A, Camici GG, Pozzi L, Cardinetti D (2011). c-Jun N-terminal kinase regulates soluble Aβ oligomers and cognitive impairment in AD mouse model. J Biol Chem.

[b37] Seki E, Brenner DA, Karin M (2012). A liver full of JNK: signaling in regulation of cell function and disease pathogenesis, and clinical approaches. Gastroenterology.

[b38] Shoji M, Iwakami N, Takeuchi S, Waragai M, Suzuki M, Kanazawa I (2000). JNK activation is associated with intracellular beta-amyloid accumulation. Brain Res Mol Brain Res.

[b39] Spigolon G, Veronesi C, Bonny C, Vercelli A (2010). c-Jun N-terminal kinase signaling pathway in excitotoxic cell death following kainic acid-induced status epilepticus. Eur J Neurosci.

[b40] Suckfuell M, Canis M, Strieth S, Scherer H, Haisch A (2007). Intratympanic treatment of acute acoustic trauma with a cell-permeable JNK ligand: a prospective randomized phase I/II study. Acta Otolaryngol.

[b41] Touchard E, Omri S, Naud MC, Berdugo M, Deloche C, Abadie C (2010). A peptide inhibitor of c-Jun N-terminal kinase for the treatment of endotoxin-induced uveitis. Invest Ophthalmol Vis Sci.

[b42] Wang J, Van DeWaterTR, Bonny C, de RibaupierreF, Puel JL, Zine A (2003). A peptide inhibitor of c-Jun N-terminal kinase protects against both aminoglycoside and acoustic trauma-induced auditory hair cell death and hearing loss. J Neurosci.

[b43] Zhao Y, Spigolon G, Bonny C, Culman J, Vercelli A, Herdegen T (2012). The JNK inhibitor D-JNKI-1 blocks apoptotic JNK signaling in brain mitochondria. Mol Cell Neurosci.

[b44] Zhu X, Raina AK, Rottkamp CA, Aliev G, Perry G, Boux H (2001). Activation and redistribution of c-jun N-terminal kinase/stress activated protein kinase in degenerating neurons in Alzheimer's disease. J Neurochem.

[b45] Zhuang ZY, Wen YR, Zhang DR, Borsello T, Bonny C, Strichartz GR (2006). A peptide c-Jun N-terminal kinase (JNK) inhibitor blocks mechanical allodynia after spinal nerve ligation: respective roles of JNK activation in primary sensory neurons and spinal astrocytes for neuropathic pain development and maintenance. J Neurosci.

